# Thiamine as a peripheral neuro-protective agent in comparison with N-acetyl cysteine in axotomized rats

**DOI:** 10.22038/IJBMS.2023.67157.14726

**Published:** 2023

**Authors:** Maryam Mousavinezhad-Moghaddam, Morteza Behnam-Rassouli, Narges Valizadeh, Naser Mahdavi-Shahri, Seyed Abdolrahim Rezaee

**Affiliations:** 1 Biology Department, Faculty of Science, Ferdowsi University of Mashhad, Mashhad, Iran; 2 Immunology Research Center, Inflammation and Inflammatory Diseases Division, Faculty of Medicine, Mashhad University of Medical Sciences, Mashhad, Iran

**Keywords:** Axotomy, Inflammation, Neuro-protective, N-acetyl cysteine, Thiamine

## Abstract

**Objective(s):**

In this study, the impact of thiamine (Thi), N-acetyl cysteine (NAC), and dexamethasone (DEX) were investigated in axotomized rats, as a model for neural injury.

**Materials and Methods:**

Sixty-five axotomized rats were divided into two different experimental approaches, the first experiments included five study groups (n=5): intrathecal Thi (Thi.it), intraperitoneal (Thi), NAC, DEX, and control. Cell survival was assessed in L5DRG in the 4^th^ week by histological assessment. In the second study, 40 animals were engaged to assess *Bcl-2*, *Bax*, *IL-6*, and *TNF-α *expression in L4-L5DRG in the 1^st^ and 2^nd^ weeks after sural nerve axotomy under treatment of these agents (n=10).

**Results:**

Ghost cells were observed in morphological assessment of L5DRG sections, and following stereological analysis, the volume and neuronal cell counts significantly were improved in the NAC and Thi.it groups in the 4^th^ week (*P*<0.05). Although *Bcl-2* expression did not show significant differences, *Bax* was reduced in the Thi group (*P*=0.01); and the *Bcl-2/Bax* ratio increased in the NAC group (1^st^ week, *P*<0.01). Furthermore, the *IL-6* and *TNF-α* expression decreased in the Thi and NAC groups, on the 1^st^ week of treatment (*P*≤0.05 and *P*<0.01). However, in the 2^nd^ week, the *IL-6* expression in both Thi and NAC groups (*P*<0.01), and the* TNF-α* expression in the DEX group (*P*=0.05) were significantly decreased.

**Conclusion:**

The findings may classify Thi in the category of peripheral neuroprotective agents, in combination with routine medications. Furthermore, it had strong cell survival effects as it could interfere with the destructive effects of *TNF-α* by increasing *Bax*.

## Introduction

Neurodegenerative and also neural death in accidents are major cost-consuming concerns for public health authorities, families, and the affected subjects. However, despite very strong attempts in modern medicine, the main changes are yet to be introduced to reduce the destructive effects of such diseases. 

The response to the nerve injury is followed by a series of degenerative cellular and molecular events, both in the proximal stump referred to as retrograde degeneration (1), and in the distal stump, which is called Wallerian degeneration (2). Retrograde degeneration induces cell death in the dorsal root ganglia (DRG) and anterior horn of the spinal cord, which are implicated in the decrement of sensory and motor functions of peripheral nerves (3, 4). Inflammatory reactions, as a danger signal, are very complex from damage to healing processes, which may have beneficial or harmful effects (5). Schwann cells, macrophages, and lymphocytes produce pro-inflammatory cytokines such as *TNF-α*, *IL1-β*, and *IL-6* via NF-κB signaling pathway activation (6). Therefore, targeting inflammation to potentiate the healing process and reduce the destructive effects are very important for therapeutic purposes.

After nerve injury, growth factors derived from the target tissues cannot reach the cell body, and instead, new retrograde signals are transferred from the lesion site back to the neuron cell body, causing changes in gene expression and increasing the production of growth and survival factors (7); however, many conditions result in inflammation and cell apoptosis (8, 9). In the intrinsic pathway of apoptosis, the ratio of the two intermediate molecules *Bcl-2* and *Bax* at the mitochondrial level, as anti- and pro-apoptotic factors, determines the death or survival of the damaged neurons (10). Also, a group of retrograde signals that are transmitted from the lesion site to the cell body are inflammatory signals that can induce neuronal death by external pathways (11). 

Thiamine or vitamin B1 has therapeutic applications for its beneficial effects as a cofactor for some enzymes, mainly with mitochondrial localization, which is very important for preventing apoptosis (12). Thiamin is in the cationic form (T+) at physiological pH and hence affects the membrane potential, transmission, and conduction of nerve messages in neurons (13). It is phosphorylated to thiamine monophosphate (TMP), thiamine pyrophosphate (TPP), and thiamine triphosphate (TTP) (14). Unlike TTP and TPP, which are involved in energy metabolism reactions, the main and central role of T+ is the anti-oxidant role, in which, thiamine is oxidized to a form of thiamine disulfide and thiochrome compounds, acting as anti-oxidants, which can modulate inflammatory reactions and apoptosis events (15). 

Taken together, in the present study considering the potential properties of thiamine, the anti-inflammatory, anti-cell death, and morphological improvements were evaluated in the DRG, following sural nerve transection, compared with N-acetyl cysteine (NAC) and dexamethasone (DEX), to determine the potential benefits in the treatment of neurodegenerative and neural death in accidents.

## Materials and Methods

***Experimental groups and surgical procedure***

All of Seventy-five male Wistar rats (220–280 g) aged 8 to 10 weeks were enrolled in the experiments. Animals were housed on a 12 hr light/dark cycle and cared for at 22−24 °C in an animal room at the Faculty of Science of Ferdowsi University of Mashhad, Mashhad, Iran. Also, the food and water were available *ad libitum*. All experimental procedures were performed in accordance with protocols according to ARRIVE (16).

For the histological study, 30 rats were included (n=5) and organized into groups 1 to 6, in which the rats in groups 2 to 5 were anesthetized with intraperitoneal injections, using a mixture of ketamine (100 mg/kg) and xylazine (10 mg/kg). Firstly, each animal was shaved and an incision was made on the skin and muscles of the right hind limb. The sural nerve was exposed and dissected. To prevent spontaneous healing, a 3 mm segment from the distal portion of the nerve was removed; then, the muscles and skin were sutured. Rats were randomly divided into the following groups: g1.intact g2.control (Cont, normal saline) g3.N-acetylcysteine (NAC, 150 mg/kg) (17) g4.dexamethasone (DEX, 0.2 mg/kg) (18), and g5.thiamin (Thi, 50 mg/kg) (19). Drug injections were introduced daily for up to 4 weeks, then rats were sacrificed and lumbar 5 dorsal root ganglion (L5DRG) was removed and fixed in 10% formalin. 

In group 6, the intrathecal (Thi.it) group, each animal was first catheterized by a PE-10 tube (AM system, America), according to Storkson’s method (20). Then, six days after catheterization, 20 µl of 2% lidocaine was injected through the catheter to examine the proper catheter status. On day 7, the animals were subjected to nerve axotomy. Rats received daily intrathecal thiamine (1.7 mg/kg) for up to 4 weeks. Since the rat’s blood volume is 30 times of cerebrospinal fluid (21), we considered the intrathecal concentration of 1.7 mg/kg as systemic 50 mg/kg. At the end of the experiment, the animals were anesthetized, and then 50 µl of toluidine blue dye was injected through the catheter. The L5DRG was pulled out about half an hour later; the bluish color of the L5DRG was considered as correct catheterization, and the ganglion was fixed in a 10% formalin solution.

In the molecular experimental approach, forty rats were randomly divided into four experimental groups (n=10): Cont, NAC, DEX, and Thi. Briefly, following anesthesia, the sural nerve of the right leg was cut. The drug injection was intraperitoneal and daily, until sampling on days 7 and 14, when five animals in each group were sacrificed. Then, the L4-L5DRG was removed and transferred into RNA later solution (Roche, Germany) and stored at -70 °C, till assessment of the expression of the target genes. Also, five rats were engaged as an intact group. 

***Histological study***

The anonymous but coded fixed L5DRG was embedded into paraffin after tissue processing and 6 µm serial sections were prepared, using a Leitz microtome (Germany) and then, stained with cresyl violet dye. Finally, stereological techniques were used to evaluate the volume and the number of cells of the L5DRG sections (22).

***RNA extraction and cDNA synthesi*** **s**

Tissue specimens were crushed in a sterile mortar and homogenized. The tissue RNA was extracted, using the RNA extraction Mini Kit (Roche, Germany) and reverse-transcribed to complementary DNA (cDNA), using the Revert Aid™ First Strand cDNA Synthesis Kit (Fermentas, Germany), according to the manufacturer’s instructions.

***Gene expression assays***

Primers were designed by Beacon Designer software (PREMIER Biosoft International, Palo Alto, CA, USA, version 7). The sequences of the primers and probes are shown in Table 1. Beta-actin was used as rat housekeeping or reference gene in *Bcl-2*, *Bax* (TaqMan Method), and *IL-6* and *TNF-α* (SYBR Green) expression assessment. The relative two standard curve methods were used for target and reference gene quantification by a Rotorgen Q-6000 real-time PCR machine (Qiagen, GmbH, Germany). Briefly, after cDNA synthesis, 10-time serial dilutions of standards (5 for each gene) were generated, and the relative copy number for each cDNA sample was calculated, accordingly. The Rotor-Gene software was used to analyze the standards and the unknown mRNA copy numbers. The relative quantity of each mRNA was normalized to the relative quantity of the reference gene, β-actin mRNA. Then, the relative *Bcl-2*, *Bax*, *TNF-α,* and *IL-6* expression levels for each sample were calculated by the following equation: 

Normalized Index = Copy number of the gene of interest /copy number of reference gene (β-actin) (23). 

***Statistical analysis***

Statistical analysis was performed, using SPSS version 11.5 (SPSS, Chicago, IL, USA). The distribution of each variable in study groups was analyzed, using the Kolmogorov-Smirnov test. The distribution of variables in histology analyses was normal, thus parametric tests were used. In the gene expression experiments, the distribution of variables was not normal thus, non-parametric tests were used for statistical analyses. Inferential statistical methods, including one-way ANOVA and Tukey post-test as parametric, and Mann-Whitney and Kruskal-Wallis as a non-parametric test, were used to compare the differences between study groups. The correlations between different variables were evaluated, using Spearman’s test. Results were considered statistically significant if the *P*-value≤0.05.

## Results

***Morphological assessment***

**Figure 1:**
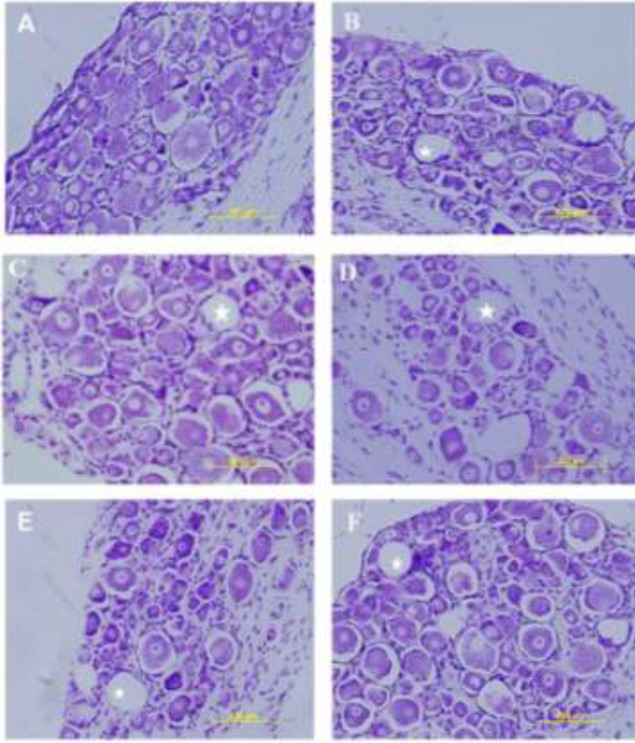
A comparison of L5DRG sections of studied animals at the end of the fourth week, after nerve transection. A) intact, B) Cont, C) NAC, D) DEX, E) Thi, and F) Thi.it. In these sections, vacuole-like structures should be apoptotic cells, which are marked with white asterisks. Magnification: 200 X

After four weeks of nerve transection, the histopathological study showed that in all of the experimental groups, some spongy form areas were observed. This phenomenon should be due to cell apoptosis and consequently, became vacuolation as ghost cells. However, in intact samples, as it was expected, such-pattern did not exist. Figure 1 shows the longitudinal sections of L5DRG stained with cresyl violet in the 4^th^ week, after nerve axotomy. 

***Morphometrical assessments***

*L5DRG volume*

**Figure 2:**
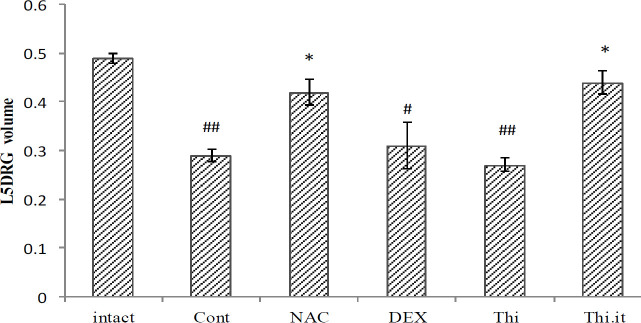
Mean of the L5DRG volume in different experimental groups (Control, Dexamethasone, N-acetylcysteine, Thiamin, Intraceal group), at the end of the fourth week, after nerve transection of the wistar rats. Data are presented as mean±SEM. *P<0.05 when compared with control group and #*P*<0.05; ##*P*≤0.01when compared with intact (n=5)

The comparison of results of the L5DRG volume between intact and control groups showed a significant decrease (*P*=0.01) in the 4^th^ week, after axotomy. The mean volume of L5DRG in different experimental groups showed that L5DRG volume is more sustainable in the NAC and Thi.it groups, compared with the control, in which the volume was decreased (*P*=0.044 and *P*=0.039, respectively); however, there were no significant differences in the other experimental groups (Figure 2). 

*L5DRG cell counts*

**Figure 3:**
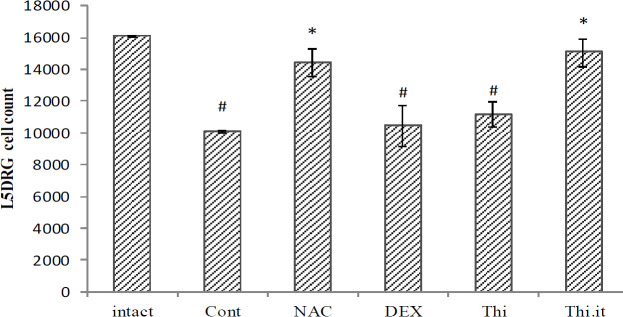
Mean of L5DRG cell number present in different experimental groups of (Control, Dexamethasone, N-acetylcysteine, Thiamin, Intraceal group). four weeks after nerve axotomy of the wistar rats. Data are presented as mean±SEM. *P<0.05 when compared with control group; #*P*<0.05 when compared with intact (n=5)

The comparison of results of the L5DRG cell counts between intact and control groups showed a significant decrease (*P*=0.023) in the fourth week, following nerve transection. The results of cell count showed significant cell survival in the number of L5DRG cells in the NAC and Thi.it groups, compared with the control group (*P*=0.043 and *P*=0.037, respectively), while there were no significant differences in the other experimental groups (Figure 3). 

***Gene expression assessments***

*Bcl-2 expression in L4-L5DRG*

**Figure 4.**
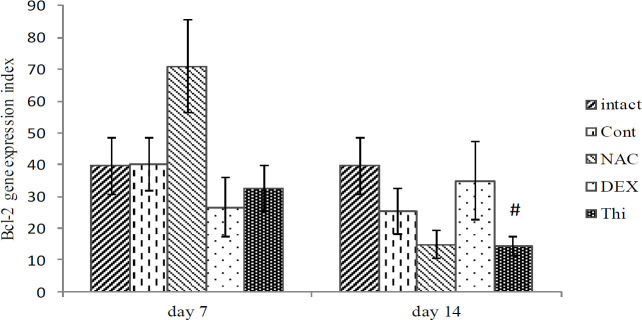
Mean expression of Bcl- 2 in L4-L5DRG in different experimental groups of (Intact, Control, Dexamethasone, N-acetylcysteine, Thiamin, Intraceal group)at the end of the first and the second weeks, after sural nerve transection of the wistar rats. Data are presented as mean±SEM. #*P*<0.05 when compared with intact (n=5)

The findings showed that even the mean *Bcl-2* expression in L4-L5DRG between intact and control groups in the 1^st^ and 2^nd^ weeks did not meet the 95% confidence interval (CI), and it was meaningful at 91% CI (*P*=0.09) in the 2^nd^ week. However, the results showed that there were no significant differences between the experimental and control groups at the end of the first and second weeks, after the sural nerve axotomy (Figure 4).

*Bax expression in L4-L5DRG*

**Figure 5:**
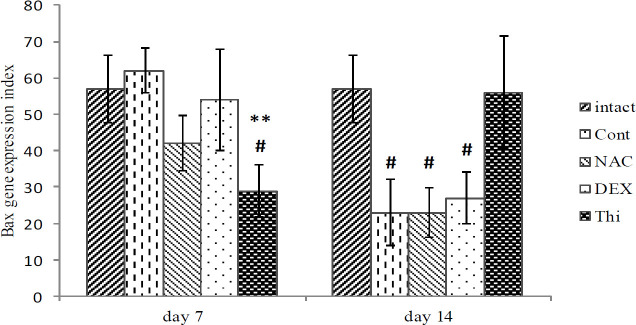
A comparison of mean expression of the *Bax* gene in L4-L5DRG in different experimental groups of Control, Dexamethasone, N-acetylcysteine, Thiamin, Intraceal group at the end of the first and the second weeks, after sural nerve transection. Data are presented as mean±SEM at the end of the first and second weeks, after nerve axotomy of the wistar rats. Data are presented as mean±SEM. **P≤0.01 when compared with control group and #*P*≤0.05 when compared with intact (n=5)

The *Bax* expression in L4-L5DRG in comparison between intact and control groups decreased significantly (*P*=0.05), in the 2^nd^ week after axotomy. Also, a statistical comparison of the results showed that *Bax* expression was significantly decreased at the end of the first week in the Thi group (*P*=0.01), whereas, in the other experimental groups the difference was not significant in comparison with the control group (Figure 5).

*Ratio of Bcl-2/Bax expression in L4-L5DRG*

**Figure 6:**
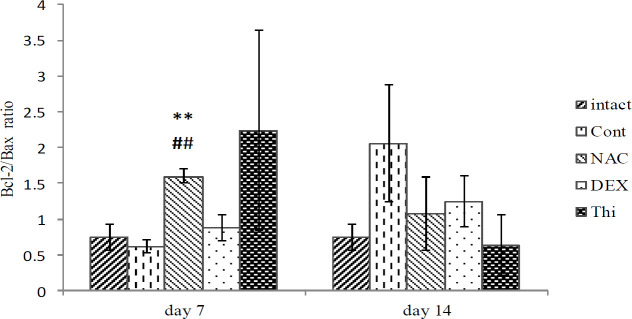
Mean expression ratio o f *Bcl-2*/*Bax* in L4-L5DRG in different experimental groups of Control, Dexamethasone, N-acetylcysteine, Thiamin at the end of the first and the second weeks, after sural nerve axotomy of the wistar rats. Data are presented as mean±SEM. **P<0.01 when compared with control group and ##*P*<0.01 when compared with intact

The findings showed that the ratio of *Bcl-2/Bax* expression in L4-L5DRG in comparison between intact and control groups in the 1^st^ and 2^nd^ weeks was not meaningful. In the NAC group, a significant increase in the *Bcl-2/Bax* ratio was seen at the end of the first week (*P*=0.007), compared with the control group, but there were no significant differences in the other experimental groups. The findings in the second week of treatment did not have any significant differences (Figure 6).

*TNF-α expression in L4-L5DRG*

**Figure 7:**
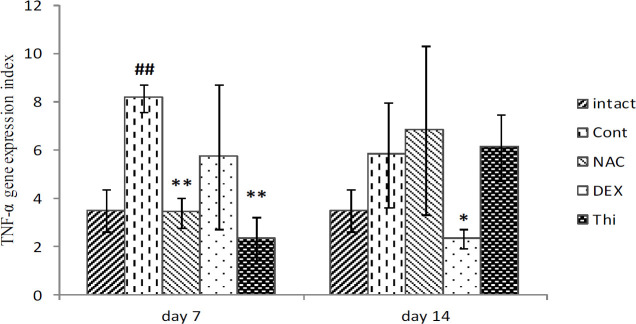
Mean expression of *TNF-α* in L4-L5DRG in different experimental groups of Control, Dexamethasone, N-acetylcysteine, Thiamin at the end of the first and second weeks, after sural nerve transection of the wistar rats. Data are presented as mean±SEM. *P≤0.05; ** *P*≤0.01 when compared with control group and ##*P*<0.01 when compared with intact (n=5)

*TNF-α* mainly has two different forms, soluble and transmembrane (tmTNF). In this study, the expression of both forms was evaluated at the site of injury. The *TNF-α* expression in L4-L5DRG in comparison between intact and control groups increased significantly (*P*=0.007), in the 1^st^ week after axotomy. Thiamine and NAC significantly suppressed the *TNF-α* expressions in the acute phase of inflammation in the axotomized site, in the 1^st ^week of treatment (*P*=0.01 and *P*=0.009, respectively). While in the second week, only DEX could significantly decrease the *TNF-α* expression (*P*=0.05) (Figure 7).

*IL-6 expression in L4-L5DRG*

**Figure 8:**
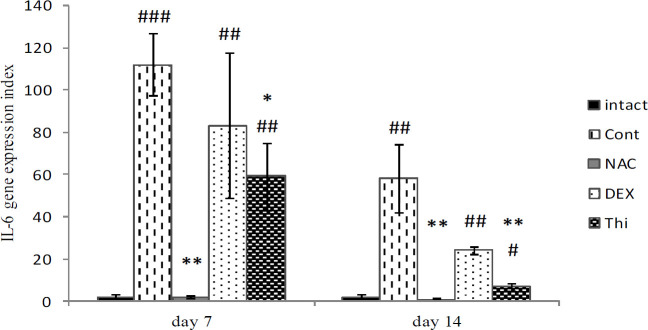
The mean expression of *IL-6* in L4-L5DRG in different experimental groups of Control, Dexamethasone, N-acetylcysteine, Thiamin at the end of the first and second weeks, after sural nerve transection of the wistar rats. Data are presented as mean ± SEM. *P≤0.05; **P<0.01 when compared with control group and #*P*<0.05; ##*P*<0.01; ###*P*≤0.001 when compared with intact (n=5)

Comparison of mean results of the *IL-6* expression in the L4-L5DRG control group and intact group showed a significant increase on days 7 and 14 after axotomy (*P*=0.001 and *P*=0.007). These results revealed a significant decrease in the *IL-6 *expression at the end of the first week in the Thi and NAC groups (*P*=0.05 and *P*=0.007, respectively) and at the end of the second week in the same groups (*P*=0.007), compared with the control group. However, the results in the DEX group showed no significant difference (Figure 8).

## Discussion

Peripheral nerve injuries result in complex cellular and molecular events that can determine the fate of healing or damage. In such a situation, several factors affect neuronal survival and death in DRG, following nerve transection, mainly necrosis and apoptosis (24). In the present study, Figure 1 shows some apoptotic areas (ghost cells), compared with the intact group, and it means that none of our treatments could prevent the complications of axotomy. However, with more precise assessment, using stereological techniques, the volume and the cell counts of the DRGs were improved in the systemic injection of NAC and intrathecal thiamine, and these treatments could prevent more apoptotic events (Figures 2 and 3). Of note, systemic thiamine administration could not overcome apoptosis but intrathecal administration could. 

Even though thiamine has anti-reactive oxygen species (ROS) activities, the systemic administration of thiamine is phosphorylated very quickly (14), and therefore, in the present study, only Thi.it could induce anti-oxidant activities. NAC, as a neuro-protective agent also ​​can inhibit cell apoptosis by glutathione reduction and decrease of ROS activity (25).

Our biomarker assessment of apoptosis demonstrated that *Bax* expression was decreased in the Thi treatment. However, the *Bcl-2/Bax* ratio, which is the index of cell survival, was reasonably improved by NAC treatment in the first week of assessments. It is well-known that cell death is progressive and widespread in DRG neurons, and the intrinsic apoptotic pathway is the main mechanism of cell death (26, 27). In this study, at least, thiamine treatment significantly reduced the *Bax* expression in the first week after injury. These findings showed that thiamine at 50 mg/kg had an anti-apoptotic effect on neurons in axotomized DRG, in a dose and time-dependent manner. Moreover, NAC treatment also can produce such effects. Furthermore, when the *Bcl-2/Bax* ratio was taken into account, this ratio was elevated in the NAC group, in the first week after axotomy, which was in favor of cell survival (Figures 4, 5, and 6).

In a study on sural axotomized DRG neurons, the *Bcl-2* expression was decreased (but not significantly) and the *Bax* expression was increased (28), while in peroneal axotomized DRG neurons, *Bax* expression was significantly increased and *Bcl-2* expression was unchanged (28). These findings suggest that the peroneal DRG neurons are affected by an upstream protective response that results in down-regulation of *Bax*, whereas this effect was absent in the sural axotomized DRG neurons (28). However, in a study by Gillardon *et al. *(10), measuring L4-L6 DRG mRNA, it was found that after sciatic nerve axotomy, *Bcl-2* expression was decreased by about 30%, whereas the change in the *Bax* expression levels was not significant. Such various results in gene expression studies may be due to the differences in the methodology or targeting of cell subpopulations (28). 

Many studies suggested that benfotiamine, a thiamine derivative, improves post-myocardial infarction and increases the Bcl-2 protein levels (29). It also prevents lipopolysaccharide (LPS)-induced apoptosis and increases the *Bcl-2* expression in a murine macrophage cell line (30). Of note, following stress induction in pericyte cells and decrement in the *Bcl-2/Bax* ratio, administration of thiamine completely reverses the deleterious effects of the injuries (31). Moreover, thiamine deficiency increases cell death and decreases *Bcl-2* expression in hybridoma cell culture (32, 33).

According to the results of the present study, although thiamine was effective in decreasing the *Bax* expression in the first week, its effect on the *Bcl-2/Bax* ratio was not so remarkable. It seems that increasing the dose of thiamine could result in better improvements; therefore, more studies are necessary for a better understanding of its impact on neural damage (Figures 4, 5, and 6).

In this context, it has been reported that NAC decreases neural death in L4-L5DRG after sciatic nerve axotomy (33) and can significantly increase the *Bcl-2/Bax* ratio by increasing *Bcl-2* and decreasing *Bax* expression (28), with strong anti-oxidant properties (34). In the present study after the NAC treatments, a significant increase in the *Bcl2/ Bax* ratio on day 7 was observed, of course without a significant effect on *Bcl2 *and *Bax*, and surprisingly the ratio was in favor of cell survival. The absence of such an increase in the second week is probably partly due to the method of sampling; because DRGs contain a mixture of cells, including neurons, glial cells, Schwann cells, and macrophages, and the whole population of the cells in tissue should be considered for analysis (Figures 4, 5, and 6), but the sural neurons make up less than half the population of DRG cells (10). Of note in the other studies, these damaged cells in NAC treatment had a stronger response in the first week, compared with the second week (28). 

In our study, DEX was used as an anti-inflammatory control treatment, which also can affect apoptosis events. Different studies have reported different effects of DEX on the expression of *Bax* and *Bcl-2 *and other molecules in the Bcl family (35-38). This anti-inflammatory factor is known as an anti-apoptotic factor and at high doses, as a pro-apoptotic agent (39). 

Neurons, satellite glial cells (SGCs), Schwann cells, and immune cells in the DRG are implicated in inflammatory responses, following peripheral nerve injuries. Furthermore, in the inflammatory reactions, many cytokines and signaling pathways are involved such as *TNF-α*, *IL1-β,* and *IL-6* as pro-inflammatory cytokines, and NF-κB and inflammasome pathways, as signaling events (6). One of the other sides of neurodegenerations is inflammatory cytokines, such as *TNF-α* and *IL-6* which are specifically assessed here. Thiamine and NAC significantly suppressed the *TNF-α* expressions in the acute phase of inflammation in the axotomized site on the 1^st ^week of treatment. While in the second week, only DEX significantly decreased the *TNF-α* expression (Figure 7).

*TNF-α* plays a critical role in inflammatory neurodegenerative diseases. The studies on neurodegenerative diseases demonstrated that in the acute phase of inflammation, *TNF-α* has been complicated in the demyelinating events. While during the late phase of the crisis, it has immunosuppressive activity. Therefore, it seems that both thiamine and NAC had improving activities on the acute phase of the axotomized site (Figure 7).

*TNF-α* has two biologically different receptors TNFR-1 and 2, which differ in structure, ligand affinity, cell expression, and signaling pathways. Systemic secretion or the local soluble form of *TNF-α* has a higher affinity for TNF-R1, promoting inflammatory reactions, and resulting in necrosis, apoptosis, vascular leak, and therefore, leading to thrombosis (40). On the other hand, tm-*TNF-α *binds mainly to TNFR2 and induces immunosuppressive activities, consequently overcoming inflammation, and resulting in cell survival (40). In transgenic mice, tm-*TNF-α* inhibits the development and progression of experimental autoimmune encephalitis (EAE) (41). Additionally, Ohtori *et al.*, showed that following sciatic nerve lesions in mice, *TNF-α* in SGCs, and *TNF-α* receptors in both SGCs and DRG neurons were increased (42). Other studies have also suggested that following chronic constriction injury (CCI) in mice, *TNF-α* mRNA expression in DRG increased by about 2.5-fold in the first to third days after the lesion, and then, decreased in the 7^th^ and 14^th^ days (43, 44). Similarly, sciatic nerve transection increased *TNF-α* in L4-L5DRG neurons, and SGCs on the 7^th^ and 14^th^ days, and the day 7 results were more severe than those of day 14 (45).

*IL-6* also like *TNF-α* has pro-inflammatory and anti-inflammatory properties in tissue injury. The results of the present study demonstrated that thiamine significantly decreased the expression of both potent pro-inflammatory cytokines, *TNF-α* and *IL-6,* after sural nerve transection. Therefore, it can be concluded that the anti-inflammatory effects of thiamine are more potent than its anti-oxidant effects, and thus can reduce extrinsically and maybe cell death pathways in L4-L5DRG cells (Figures 7 and 8).

The findings of different treatments on L4-L5DRG of this study showed that administration of thiamine and NAC had a significant effect on decreasing the *IL-6* expression in both time intervals after axotomy (Figure 8). *IL-6* is involved in both inflammatory and anti-inflammatory reactions and is expressed at very low levels in normal DRGs (46). It is assumed that *IL-6* up-regulation in neurons is associated with the increased expression of neural growth factors, consequently, with axonal growth (47), in the late phase of inflammation. Additionally, some published reports demonstrated that *IL-6* levels in rat DRG increased in the second and fourth days (46) or the seventh day after sciatic nerve transection (48). Also, it has been shown that *IL-6* mRNA and protein levels in L4-L5DRG are increased on days 3 and 14, after sciatic nerve lesion (49). 

Benfotiamine, a derivative of thiamine, has potent properties, reducing inflammation at the site of injury; for example, it has been shown that it inhibits the pro-inflammatory impacts of *IL-6* and *TNF-α* in microglial cells, following stimulation with LPS, which is mediated by a decrease in NF-κB (50). Furthermore, thiamine deficiency highly increases inflammatory cytokines such as *IL-1*, *IL-6*, and *TNF-α* (51). 

The results of our study showed that NAC also reduced the *TNF-α* and *IL-6* expression, after axotomy. Therefore, NAC has strong anti-oxidant and anti-inflammatory properties that can affect both the intrinsic and extrinsic pathways of apoptosis, thereby, reducing the death of L4-L5DRG neurons (Figures 7 and 8).

Similarly, studies have shown that LPS administration in mice is associated with increased ROS and inflammatory factors, and administration of NAC inhibits NF-κB translocation to the nucleus and reduces levels of ROS and *TNF-α* in macrophages and lymphocytes (52, 53). 

DEX has potent anti-inflammatory properties and decreases the activity of immune cells and reduces the production of inflammatory mediators (54, 55). Although the results of the present study showed that DEX partially reduced *TNF-α,* not *IL-6* expression, compared with the control group (Figures 7 and 8), which probably was due to an insufficient dose of DEX. It has been suggested that glucocorticoids such as DEX, which are used widely in neural damage because of their potent anti-inflammatory effects (56), have been less effective in our study, compared with NAC and thiamine. In our study, the systemic administration of DEX did not prevent the reduction of ganglion volume, the number of L5DRG neurons, or apoptosis (Figures 2−6). However, it should be noted that due to the severe catabolic effects, higher doses of DEX are not feasible in the rat model because of its toxic effect and animal death. Since this study is a pilot, we had some limitations. The main limitation of this study is the lack of nerve function tests to support our hypothesis, and the authors highly recommend applying tests that can clarify the nervous function besides other works. 

**Table 1:** Primers designed for semi-quantitative real-time polymerase chain reaction of *Bcl-2*, *Bax*, *TNF-α*, *IL-6*, and *β-actin*

Gene	Primers sequences
Bcl-2	Forward: 5'- AGG ATA ACG GAG GCT GGG ATG-3' Reverse: 5'- CTC ACT TGT GGC CCA GGT ATG-3'
Bax	Forward: 5'- CAT CAG GGT TTC ATC CAG GAT C-3' Reverse: 5'- CCA CAT CAG CAA TCA TCC TCT G-3'
TNF-α	Forward: 5'-GAG TCA TTG CTC TGT GAG-3' Reverse: 5'-CTC TGA GGA GTA GAC GAT A-3'
IL-6	Forward: 5'-GCC CTT CAG GAA CAG CTA TGA-3' Reverse: 5'-TGT CAA CAA CAT CAG TCC CAA AGA-3'
β -actin	Forward: 5'-CCC GCG AGT ACA ACC TTC T-3' Reverse: 5'- CCA TCA CAC CCT GGT GCC TA-3'

## Conclusion

Thiamine can also, like NAC, have anti-oxidant properties and increase catalase and glutathione levels; therefore, it might have anti-inflammatory and anti-apoptotic activities. The histological analysis in our study showed that systemic thiamine infusion is not able to overcome apoptosis, despite decreasing *Bax* pro-apoptotic factor in DRG cells; however, the intrathecal administration had an anti-apoptotic effect like NAC and could affect the volume and the cell counts in the DRG. Therefore, thiamine in a dose and time-dependent manner had anti-inflammatory and anti-apoptotic effects, particularly if it can be injected intrathecally. The low efficiency of neuroprotective activities of thiamine in systemic administration can be attributed to the inadequate dose and its quick phosphorylation in the body fluids. Therefore, more efforts for chemical changes of thiamine are necessary to upraise its long time effects in systemic administration, without changes in functional activities to be used in neurodegenerative diseases, since it has fewer side effects, compared with the other drugs. 

## Authors’ Contributions

M MM handled practical works and prepared the draft; M BR planned and supervised the physiological works; N V performed molecular techniques; N MS performed histological analysis; SA R planed the molecular techniques, supervised, and compiled the manuscript.

## Ethical Approval

This work does not contain any studies with human participants. It was approved by the biomedical ethics committee of Ferdowsi University of Mashhad, Iran (approval ID: IR.UM.REC.1397.063).

## Consent for Oublication

The authors give their consent for the publication of identifiable details within the text to be published in the above journal and article. 

## Availability of Data and Material

The data that support the findings of this study are included in the manuscript and are available from the corresponding author upon reasonable request.

## Funding

This study was financially supported by the Vice-Chancellor for Research and Technology, Mashhad University of Medical Sciences, Mashhad, Iran, (under grant mums. 940156) and Vice-Chancellor for Research and Technology of Ferdowsi University of Mashhad (IR.UM.REC.1397.063). 

## Conflicts of Interest

There are no competing interests in this study.
